# Supplementary material for: Cognitive impairment and its risk factors among Myanmar elderly using the Revised Hasegawa’s Dementia Scale: A cross-sectional study in Nay Pyi Taw, Myanmar
Source: PLoS One. 2020 Jul 28;15(7):e0236656. doi: 10.1371/journal.pone.0236656 (PMC7386565; doi:10.1371/journal.pone.0236656)
Supplement: Attachment — Submitted filename: Response to Reviewers.docx [file pone.0236656.s003.docx]

**Response letter (**Response to Reviewers**)**

**PONE-D-20-03550: Cognitive impairment and its risk factors among Myanmar elderly using the Revised Hasegawa’s Dementia Scale: A cross-sectional study in Nay Pyi Taw, Myanmar**

**Thank you very much editor and reviewers for your valuable comments and suggestions. We have revised the manuscript accordingly. The revised and edited sentences (and words) are mentioned using a track-changes function in the revised manuscript. We also submitted a clean version of revised manuscript as a separate file. In below responses, we noted reviewer’s comments in black color and our responses in blue color.**

**Reviewer #1:**

**Reviewer #1:** All comments have been addressed.

**Authors’ response:** Thank you very much for your insightful comments and suggestions on our manuscript, which has significantly improved by your constructive comments and suggestions.

**Reviewer #2:**

**Reviewer #2:** The manuscript is much improved from the first round, though the authors still need to address a few issues.

**Authors’ response:** Thank you very much for your careful reading of our manuscript and providing valuable comments and suggestions, which have been very helpful in improving our manuscript. We have revised our manuscript according to your comments and suggestions point by point.

**Q-1.** I am still unclear by what the authors mean when they write 'unable to subtract the value'. Please explain, in the manuscript, what you mean by 'value'. Are you referring to the ability to perform simple arithmetic?
**A-1: Authors’ response:** Thank you very much for your comments. Yes, we would like to mention the ability to perform simple arithmetic calculation. The participant needs perform serial subtractions of 7s. The question is "subtract 7 from 100". As suggested, we revised our pervious sentences “*Elderly individuals who lived in the study area less than six months, who were diagnosed with cognitive impairments along with mental and physical disorders (seriously ill), and who did not understand the Myanmar language and were unable to subtract the value were excluded from the study*.” **to read as follows:** “Elderly individuals who lived in the study area less than six months, those who were diagnosed with cognitive impairments along with mental and physical disorders (seriously ill), those who did not understand the Myanmar language, and those who were unable to perform simple arithmetic calculation were excluded from the study.”  **[Methods, Line 135-139, Page 6]**

**Q-2.** The authors' response to my question about why they stratified the HDS-R should be added to the manuscript.
**A-2: Authors’ response:** Thank you very much for your comments. As suggested, we added information of why we stratified the HDS-R in the manuscript as follows: “The dependent variable was cognitive impairment, which was dichotomized into “≤20 points” (presence of cognitive impairment) and “≥21 points” (absence of cognitive impairment). The cut-off point, 20/21, was applied based on the evaluation study of HDS-R test reporting 0.90 for sensitivity and 0.82 for specificity [42]. **[Methods, Line 168-171, Page 7-8]**

**Q-3.** The chi-square critical values in Table 2 do not communicate useful information. Please report the exact p-values instead.
**A-3: Authors’ response:** Thank you very much for your comments. We have reported exact p-values in the Table 1 and Table 2. **[Results: Table 1, Page 10-11; Table 2, Page 12]**

**Q-4.** Since the study is cross-sectional, the authors cannot write about 'risk'. In the second paragraph of the discussion, they should replace 'risk' with 'odds'. The same edit should be made in other areas of the manuscript where the term risk may occur.
**A-4: Authors’ response:** Thank you very much for your valuable comments. We have replaced 'risk' with 'odds' throughout the revised manuscript. **[Discussion, Line 279, Page 17; Line 301, Page 18; Line 325, Page 19; Line 347, 358, Page 20; Line 363, Page 21]**

**Q-5.** Please explain how missing data were handled in the regression analysis.
**A-5: Authors’ response:** Thank you very much for your comments. During data cleaning stage, we excluded 54 participants/cases those who missed to answer the dependent and independent variables. As suggested, we amended pervious sentence “*After cleaning the data and removing those with missing responses to the main outcome variables, 757 elderly participants (males: 246 [32.5%], females: 511 [67.5%]) were considered for the final data analysis*.” **to read as follows:** “After cleaning the data and removing those with missing responses to the dependent and independent variables, 757 elderly participants (males: 246 [32.5%], females: 511 [67.5%]) were considered for the final data analysis.” **[Methods, Line 154-157, Page 7]**

**Q-6.** In the limitations section, the authors write that domain-specific cognitive impairment could not be assessed because a psychiatrist did not assess the participants. However, various cognitive tests exist to permit the assessment of specific cognitive domains, so the authors should say that the HDS-R allowed them to assess global cognitive impairment, and they were unable to measure impairment in specific domains.
**A-6: Authors’ response: Thank you very much for your comments.** As suggested, **we** revised the previous sentence from limitation section “*However, cognitive impairment among the participants in this study was not confirmed by a psychiatrist; therefore, cognitive impairment in specific domains such as executive function, spatial working memory, processing speed, attention, verbal memory, or verbal fluency could not be ruled out*.” **to read as follows**: “However, cognitive impairment among the participants in this study was not confirmed by a psychiatrist. Therefore, cognitive impairment in specific domains such as executive function, spatial working memory, processing speed, attention, and verbal memory or verbal fluency could not be ruled out regardless of HDS-R allowance to assess cognitive impairment.” **[Discussion-limitation, Line 393-397, Page 22]**

**Q-7.** I am uncertain of the value of figure 1 - suggest deletion.
**A-7: Authors’ response:** Thank you very much for your comments. As suggested, we deleted figure1.

**Q-8.** many of the odds ratios in Table 4 are still fairly wide, despite the large sample size. The authors should add mention of this fact to the limitations.
**A-8: Authors’ response:** Thank you very much for your valuable comments. As suggested, we have added below sentence to limitation “Furthermore, the fairly wide confidence intervals were observed for some predictors despite the large sample size.” **[Discussion-limitation, Line 397-398, Page 22]**

**Q-9.** At the end of the Ethical considerations section, the authors should mention that they reported their study in conjunction with the STROBE guidelines. Please cite the guidelines and include the STROBE checklist as an appendix.

**A-9: Authors’ response:** Thank you very much for your valuable suggestion. As suggested, at the end of the Ethical consideration section, we added STROBE checklist information as follows: “Furthermore, this study followed the Strengthening the Reporting of Observational Studies in Epidemiology (STROBE) guidelines (Appendix 1).” **[Methods, Line 212-213, Page 9]**

**Newly added reference**

1. Imai Y, Hasegawa K. The revised Hasegawa’s Dementia Scale (HDS-R)- Evaluation of its usefulness as a screening test for dementia. J Hong Kong Coll. Psychiatr .1994; 4:20-24.

**Additional revision**

1. We have corrected HSR-D to HDS-R in introduction section. **[Introduction, Line 116-117, Page 5]**
2. We amended the journal title abbreviations of reference no. 10,12,13, and 16.
